# Biomarkers of acute kidney injury and associations with short- and long-term outcomes

**DOI:** 10.12688/f1000research.7998.1

**Published:** 2016-05-24

**Authors:** Jennifer A. Schaub, Chirag R. Parikh

**Affiliations:** 1 Program of Applied Translational Research, Yale University, New Haven, CT, USA

**Keywords:** acute kidney injury, AKI, biomarkers, chronic kidney disease, CKD

## Abstract

Acute kidney injury is strongly associated with increased mortality and other adverse outcomes. Medical researchers have intensively investigated novel biomarkers to predict short- and long-term outcomes of acute kidney injury in many patient care settings, such as cardiac surgery, intensive care units, heart failure, and transplant. Future research should focus on leveraging this relationship to improve enrollment for clinical trials of acute kidney injury.

## Introduction

Acute kidney injury (AKI) is a common complication, occurring in about 5% of hospitalizations, and is often associated with various short- and long-term complications ^1^. AKI has been associated with short-term outcomes, such as increased length of hospital stay, length of ventilation, and in-hospital mortality ^2^. A meta-analysis of observational studies demonstrated that survivors of AKI had a long-term mortality rate of more than twice that of patients without AKI ^3^. Furthermore, increasing duration of AKI was positively associated with long-term mortality ^4, 5^. Although both the magnitude and length of AKI are significantly associated with reduced long-term survival, no clear causal relationship between AKI and mortality has been established yet. However, mechanisms are currently being elucidated, and there is increasing evidence that AKI can cause distant organ injury, including lung and cardiac injury ^6^. Despite the relationship between AKI and adverse clinical outcomes, clinical trials investigating novel treatments for AKI have not demonstrated any benefit ^7– 11^.

While increased mortality is a well-documented complication of AKI, evidence from basic science and clinical literature suggests that there may be other consequences, including increased risk for development of hypertension, increased risk of progression to chronic kidney disease (CKD), and increased risk of cardiovascular events. Survivors of AKI are about 20 percent more likely to develop elevated blood pressure after an AKI event ^12^. Rat models have shown that there is rarefaction of peritubular capillaries after an episode of AKI, which leads to salt-sensitive hypertension ^13, 14^. A meta-analysis of observational data demonstrates that AKI increases the risk of CKD by almost eightfold ^15^. Cell cycle arrest in the gap (G)2/mitotic (M) phase checkpoint during proximal tubule injury activates fibrotic cellular signals, such as transforming growth factor-β and connective tissue growth factor, which may contribute to the increased risk of CKD ^3, 15– 18^. Epidemiologic data show that AKI may increase the risk of subsequent cardiovascular events by 1.5- to 2-fold ^3^. While the definite mechanism is not clear, there is evidence that ischemia-reperfusion increases apoptosis in the heart, macrophage infiltrate, and angiotensin-converting enzyme expression ^19– 21^. There is also evidence that ischemia-reperfusion injury alters the affinity of the calcium receptor in the heart for inotropic agents ^22^.

## Potential uses of AKI biomarkers

**Figure 1.**
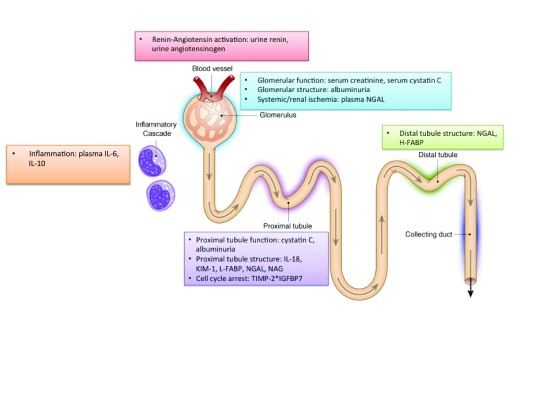
Physiology of biomarkers of AKI. Adapted from 73. AKI, acute kidney injury; H-FABP, heart fatty acid binding protein; IGFBP-7, insulin-like growth factor binding protein 7; IL-6, interleukin-6; IL-10, interleukin-10; IL-18, interleukin-18; KIM-1, kidney injury molecule-1; L-FABP, liver fatty acid binding protein; NAG, N-acetyl-β-D-glucosaminidase; NGAL, neutrophil gelatinase-associated lipocalin; TIMP-2, tissue inhibitor metalloproteinase-2.

Novel biomarkers of AKI are recent developments and typically reflect a specific component of AKI pathophysiology, including tubular injury, cell cycle arrest, systemic inflammatory pathways, and glomerular filtration ( Figure 1). Biomarkers of AKI will hopefully have several uses in clinical care, including early diagnosis of AKI, prediction of clinical outcomes, and prediction of response to therapy. Initial investigations of biomarkers of AKI sought to determine if they could diagnose AKI earlier than serum creatinine, as it often takes 2 to 3 days before serum creatinine is elevated after a renal insult. Early diagnosis would be beneficial, as it can identify a window where clinicians could potentially intervene, such as by withdrawing nephrotoxic agents or providing treatment while injury is ongoing. If effective treatments for AKI are developed, clinicians could also potentially serially follow biomarkers of injury to determine if patients are responding to treatment. Evidence has also accumulated that biomarkers of AKI are associated with various patient outcomes, which is the focus of this review.

## Biomarkers of AKI and prognosis

**Figure 2.**
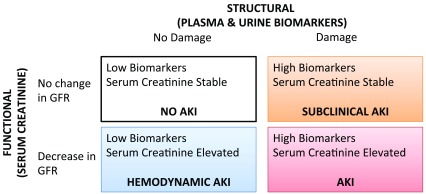
Structural *versus* functional classification of AKI. Adapted from 74. AKI, acute kidney injury; GFR, glomerular filtration rate.

Many of the novel biomarkers of AKI are intricately involved in its pathogenesis, so it logically follows that they could be associated with adverse outcomes after AKI ^23– 26^. Biomarkers of AKI have been related to short- and long-term adverse outcomes in various patient care settings. The most commonly studied short-term outcomes are in-hospital mortality, need for renal replacement therapy (RRT), and length of stay. While there is evidence that biomarkers of AKI are related to long-term mortality, data are lacking to suggest that biomarkers of AKI are related to other important long-term patient outcomes, such as cardiovascular events and CKD, although prospective studies are ongoing ^27^. Since AKI independently increases the risk of subsequent cardiovascular events and CKD, it follows that diagnostic biomarkers of AKI will also be associated with these poor outcomes. However, some AKI biomarkers may yield additional prognostic information beyond that provided by the AKI event itself. Long-term follow-up studies have identified a subgroup of patients who suffer from “subclinical AKI”. While these patients do not have AKI as defined by serum creatinine, they have elevated biomarkers of tubular injury and fare worse than patients without elevated biomarkers of AKI ^28, 29^. This suggests that biomarkers of AKI may provide additional prognostic information beyond that offered by serum creatinine. This observation has generated a new proposed framework for organizing AKI: structural *versus* functional AKI ( Figure 2). This framework highlights two groups: the aforementioned patients with subclinical AKI and those with AKI as defined by serum creatinine but without any structural damage as indicated by low tubular injury markers, or “hemodynamic” AKI. This could prove meaningful, as it suggests that clinical trials investigating treatments for conditions such as acute tubular necrosis are enrolling patients unlikely to experience benefit (those with hemodynamic AKI) and failing to enroll patients who may benefit (those with subclinical AKI).

## Utility of novel biomarkers in various patient settings

While novel biomarkers of AKI reflect the same pathophysiology regardless of the patient care setting, challenges for interpreting biomarkers of AKI differ between patient care settings, including cardiac surgery, critically ill, cardiorenal syndrome, transplant, and hospitalized patients.

### Cardiac surgery

Cardiac surgery is one of the most popular settings for the evaluation of biomarkers of AKI, partially because there is a clear point of injury. AKI after cardiac surgery has been commonly ascribed to an inflammatory cascade, although thus far interventions to quell the inflammatory cascade have been unsuccessful ^10^. Biomarkers of AKI could help identify patients at higher risk of adverse outcomes.

***Short-term outcomes.*** One of the most carefully studied cohorts for biomarkers, Translational Research Investigating Biomarker Endpoints of AKI (TRIBE-AKI), featured over 1200 patients who underwent cardiac surgery. Levels of post-operative tubular injury biomarkers, such as urinary interleukin (IL)-18, neutrophil gelatinase-associated lipocalin (NGAL), kidney injury molecule-1 (KIM-1), and liver fatty acid binding protein (L-FABP), were associated with increased risk of in-hospital death, increased length of stay, and need for RRT ( Table 1) ^30, 31^. Patients with elevated urinary cystatin C, another tubular injury biomarker, additionally had increased risk of requiring RRT ^32^.

**Table 1.** Biomarkers of AKI and outcomes in cardiac surgery.

Biomarkers	OUTCOMES						
	Short-term (1 Month) outcomes				Three year mortality		
	LOS - ICU	LOS - Hospital	AKI recovery	Mortality or RRT	AKI	No AKI	Overall
**Urine IL-18** TRIBE-AKI ^28, 30^	Q1: 2.8 d (SD 6)* Q5: 5.3 d (SD 15.3)	Q1: 6.7 d (SD 6.7)* Q5: 12.2 d (SD 18)		Q1: 0.4%* Q5: 5.8%	HR 3.16 (1.53, 6.53)	HR 1.23 (1.02, 1.48)	
**Urine NGAL** TRIBE-AKI ^28, 30^	Q1: 2.7 d (SD 2.6)* Q5: 5.4 d (SD 15.9)	Q1: 6.4 d (SD 3.1)* Q5: 12.2 d (SD 18)		Q1: 0.4%* Q5: 5.4%	HR 2.52 (1.86, 3.42)	HR 0.9 (0.5, 1.63)	
**Urine L-FABP** TRIBE-AKI ^30, 31^	Q1: 2 d (IQR: 1, 3)* Q5 2.0 d (IQR: 1, 4)	Q1: 6 d (IQR: 5,8)* Q5: 7 d (IQR: 6, 10)		Q1: 1.7%§ Q5: 5.0%	HR 2.35 (1.64, 3.37)	HR 0.65 (0.45, 0.92)	
**Urine KIM-1** TRIBE-AKI ^30, 31^	Q1: 2 d (IQR: 1, 3) ^§^ Q5: 2 d (IQR: 1, 3)	Q1: 6 d (IQR: 5, 7)* Q5: 7 d (IQR: 5, 9)		Q1: 1.3%* Q5: 5.4%	HR 2.10 (1.31, 3.10)	HR 1.83 (1.44, 3.33)	
**Blood IL-6** TRIBE-AKI ^36^							HR 1.4 (0.65, 2.90)
**Blood IL-10** TRIBE-AKI ^36^							HR 0.72 (0.56, 0.93) **↑**NGAL: 0.51 (0.36, 0.73)
**IL-18 and KIM-1** Arthur 2014 ^33^				AUC 0.93(0.80, 0.98)			
**Urine angiotensinogen** Alge 2013 ^34^				AUC 0.75(0.65, 0.85)			
**Urine Renin** Alge 2013 ^34^				AUC 0.70(0.57, 0.83)			
Urine TIMP-2*IGFBP7 Meersch 2014 ^35^			AUC 0.79(0.65, 0.92)				

*p-value for trend significant, §p-value for trend not significant. All values in ( ) are 95% confidence intervals, unless otherwise noted. AKI, acute kidney injury; AUC, area under the curve; HR, hazard ratio (3 ^rd^ tertile compared to 1 ^st^ tertile); IGFBP7, insulin like growth factor binding protein 7; IL-6, interleukin-6; IL-10, interleukin-10; IL-18, interleukin-18; IQR, interquartile range; KIM-1, kidney injury molecule-1; L-FABP, liver fatty acid binding protein; LOS, length of stay; NGAL, neutrophil gelatinase-associated lipocalin; Q1, quintile 1; Q5, quintile 5; RRT, renal replacement therapy; SD, standard deviation; TIMP-2, tissue inhibitor of metalloproteinases-2; d, days.

Another cohort of cardiac surgery patients, Southern Acute Kidney Injury Network (SAKInet), were evaluated to determine whether combinations of biomarkers could predict if patients with stage I AKI (AKIN) after cardiac surgery progressed to stage III AKI or death. Urinary IL-18 combined with percentage change in serum creatinine or urinary KIM-1 had the best discriminative ability to identify patients at high risk for progressing to higher stages of AKI or death within 30 days ^33^. The SAKInet cohort study additionally evaluated urinary angiotensinogen and renin, biomarkers of intra-renal renin-angiotensin system activity, and found that they were associated with severe AKI or death within 30 days ^34^.

Changes in urinary biomarkers have been associated with improved patient outcomes. One study examined biomarkers that induce cell cycle arrest in cardiac surgery by examining the product of tissue inhibitor metalloproteinase 2 (TIMP-2) and insulin-like growth factor binding protein 7 (IGFBP-7). Differences in the decline of these biomarkers within 24 hours after surgery discriminated between patients who recovered and those who failed to recover from AKI ^35^.

***Long-term outcomes.*** In the TRIBE-AKI study, patients with AKI as defined by serum creatinine and elevated peak post-operative tubular injury markers, including urinary IL-18, NGAL, KIM-1, and L-FABP, had an increased risk of long-term mortality ^28^. Patients without AKI as defined by serum creatinine, elevated urinary IL-18, and KIM-1 also had an increase in long-term mortality of about 1.5-fold. These patients may have experienced subclinical AKI that was not detected by serum creatinine, leading to increased mortality, or it may be that urinary IL-18 or KIM-1 is capturing a systematic inflammatory process which increases the risk of death independently of AKI.

Biomarkers that probe inflammatory pathways involved in AKI have additionally been investigated in TRIBE-AKI. Elevated first post-operative serum IL-10, which is known to attenuate the inflammatory cascade, was found to decrease the risk of long-term mortality ^36^. Interestingly, this effect was isolated to patients with elevated urinary markers of tubular injury, which suggests that IL-10 protected those patients who were experiencing proximal tubular cell damage.

### Critical illness

AKI is a common complication in critical illness and is consistently associated with high mortality, although this is a challenging setting in which to study biomarkers of AKI ^1^. There may be multiple renal insults, and the timing of renal insults is not always clear. Regardless, biomarker data have identified subphenotypes for complex intensive care unit (ICU) syndromes, such as acute respiratory distress syndrome (ARDS), which benefit from different treatment strategies ^37^. The incorporation of prognostic information from AKI biomarkers could eventually lead to similar benefit. Multiple urinary biomarkers have been studied in critically ill patients and are associated with adverse outcomes, although the data are strongest for short-term outcomes ( Table 2).

**Table 2.** Biomarkers of AKI and outcomes in critically ill patients.

Biomarkers	SHORT-TERM (IN-HOSPITAL) OUTCOMES			
	Recovery of AKI	RRT	RRT or mortality	Mortality
**Urine IL-18** VALID ^38^ Doi 2011 ^42^ ARDS Network ^41^		OR 1.05 (0.56, 1.98)	OR 1.76 (1.19, 2.59)	OR 2.02 (1.41, 2.89) AUC 0.83 (0.68, 0.91) OR 2.32 (1.2, 4.4) §
**Urine NGAL** VALID ^39, 40^ Doi 2011 ^42^ ATN Trial ^44^	AUC 0.70 (0.55, 0.84)	OR 2.60 (1.55, 4.35)	HR 1.38 (0.93, 1.96)	OR 1.44 (1.00, 2.07) AUC 0.83 (0.69, 0.91)
**Urine L-FABP** Doi 2011 ^42^ VALID ^39^			HR 1.15 (0.82, 1.61)	AUC 0.90 (0.84, 0.94)
**Urine NAG** Doi 2011 ^42^				AUC 0.66 (0.50, 0.80)
**Urine Cystatin C** Nejat 2010 ^43^				OR 1.60 (1.16, 2.21) †
**Urine HGF** ATN Trial ^44^	AUC 0.74 (0.53, 0.94)			
**Urine TIMP2xIGFB7** Sapphire ^45^			HR 2.16 (1.32, 3.53)* §	
**Urine Albumin** Doi 2011 ^42^				AUC 0.72 (0.61, 0.86)

All values in ( ) are 95% confidence intervals, unless otherwise noted. *Outcomes at 9 months in patients with AKI § greater than cut-off *versus* less than cut-off † for 10-fold increase. AKI, acute kidney injury; AUC, area under the curve; HGF, hepatocyte growth factor; HR, hazard ratio (continuous unless otherwise noted); IL-18, interleukin-18; IGFBP7, insulin like growth factor binding protein 7; L-FABP, liver fatty acid binding protein; NAG, N-acetyl-beta-D-glucosaminidase; NGAL, neutrophil gelatinase-associated lipocalin; OR, odds ratio; TIMP-2, tissue inhibitor of metalloproteinases-2.

***Short-term outcomes.*** One ICU cohort study, Validation of Biomarkers for Acute Lung Injury Diagnosis (VALID), comprising critically ill patients in both medical and surgical ICU settings, collected urinary biomarker specimens on admission and 48 hours after admission in over 400 patients. In VALID, admission levels of urinary IL-18 were independently associated with the composite outcome of 28-day mortality or dialysis even after adjustment for APACHE-II score, serum creatinine, and sepsis criteria ^38^. Despite the relationship between urinary IL-18 and the composite outcome, other tubular injury markers, such as urinary NGAL and L-FABP, were associated only with the initiation of dialysis at 28 days and were not associated with the composite outcome ^39, 40^. This persisted despite the authors excluding patients with unidentified CKD or AKI upon enrollment into the study ^39^. Similar results for urinary IL-18 and NGAL have been found in other cohorts of critically ill patients. A *post hoc* case-control study of 132 patients in the ARDS Network additionally found that increasing levels of urinary IL-18 on admission were associated with increased 1-month mortality ^41^. Another prospective cohort of 339 patients in a mixed ICU setting showed that admission urinary L-FABP, IL-18, and NGAL could discriminate between survivors and non-survivors, and a combination of urinary NGAL and L-FABP was best able to discriminate between survivors and non-survivors ^42^. Additionally, increasing levels of urinary cystatin C, a biomarker of glomerular filtration, in a group of 444 ICU patients were associated with increased risk of 1-month mortality ^43^.

As in cardiac surgery, a decline in tubular injury biomarkers is also associated with improved patient outcomes. A *post hoc* analysis of 76 patients from the Acute Renal Failure Trial Network (ATN) trial, which evaluated the effect of dialysis dose on critically ill patients, investigated whether urinary biomarkers were associated with recovery from AKI and found that decreasing levels of urinary NGAL and hepatocyte growth factor (HGF) over a 2-week period predicted an increased chance of recovery of renal function 60 days after ICU admission ^44^.

***Long-term outcomes.*** While biomarkers of tubular injury are studied most carefully, another cohort comprising 744 ICU patients (Sapphire) found that urinary biomarkers of cell cycle arrest (TIMP-2*IGFBP-7) were associated with a twofold increased risk of RRT and 9-month mortality, although this relationship was found only in patients with AKI as defined by serum creatinine ^45^.

### Cardiorenal syndrome

Heart failure patients commonly suffer from AKI, which is strongly associated with increased mortality ^46^. Cardiorenal syndrome, or the co-existence of AKI and heart failure, is often ascribed to hemodynamic perturbations or venous congestion. This is likely an over-simplification, as patients with cardiorenal syndrome respond differently to standard heart failure therapies, such as diuresis and inotropic support. Biomarker research has attempted to untangle the numerous phenotypes that exist within cardiorenal syndrome ^47^.

***Long-term outcomes.*** There is extensive literature surrounding long-term prognosis and biomarkers of tubular injury in heart failure patients. Tubular injury biomarkers, including urinary KIM-1, NGAL, and N-acetyl-β-D-glucosaminidase (NAG), were associated with long-term mortality and recurrent heart failure hospitalization even after accounting for glomerular filtration rate (GFR) in multiple case-control studies and cohorts ( Table 3) ^48– 50^. KIM-1 and NAG were significantly correlated with New York Heart Association class, suggesting that tubular injury may contribute to the pathophysiology of cardiorenal syndrome ^50^. While urinary biomarkers of tubular injury have been consistently associated with mortality and other adverse outcomes, plasma NGAL has not demonstrated a consistent relationship, particularly after adjustment for renal function ^51– 53^. This has also been found in cardiac surgery and is likely because plasma NGAL is renally excreted, effectively rendering it another marker of GFR ^54^.

**Table 3.** Biomarkers of AKI and outcomes in cardiorenal syndrome.

Biomarkers	LONG-TERM OUTCOMES (>1 YEAR FOLLOW-UP)		
	MACE	Mortality	Hospitalization
**Urine NGAL** Damman 2011 ^48^		HR 1.23 (1.07, 1.41)	HR 1.01 (0.90, 1.13)
**Urine NAG** Damman 2010 ^49^ Damman 2011 ^48^	HR 1.43 (1.10, 1.84) † HR 1.22 (1.10, 1.36)	HR 1.30(1.11–1.51)	HR 1.17(1.02, 1.33)
**Urine KIM-1** Damman 2010 ^49^ Damman 2011 ^48^	HR 1.13 (1.00, 1.28) HR 1.22 (1.03, 1.45)	HR 1.11 (0.98–1.26)	HR 1.11(0.98, 1.26)
**Plasma NGAL** GALLANT ^51^ COACH ^53^ Shrestha 2011 ^52^	HR 29.83 (5.43, 163.96)* § HR 1.06 (0.66, 1.84)	HR 1.44(1.22, 1.69) §	

All values in ( ) are 95% confidence intervals, unless otherwise noted. *unadjusted and 30 day follow-up, § greater than cut-off *versus* less than cut-off, † per 5U/gram Creatinine increase. AKI, acute kidney injury; HR, hazard ratio (per standard deviation increase unless otherwise noted); KIM-1, kidney injury molecule-1; MACE, major adverse cardiac events; NAG, N-acetyl-beta-D-glucosaminidase; NGAL, neutrophil gelatinase-associated lipocalin.

### Kidney transplantation

Novel urinary biomarkers have also been investigated for prognostic utility in the kidney transplant setting and can have unique applications in the transplant field. Given the persistent shortage of organs and the increasing number of patients on the transplant list, biomarkers of AKI can potentially be used to decide if a deceased donor organ is acceptable for transplant as well as improve prediction for adverse recipient outcomes, such as delayed graft function (DGF) and graft failure ( Table 4).

**Table 4.** Biomarkers of AKI and outcomes in kidney transplantation.

Urine biomarkers	OUTCOMES		
	Short-term (1 week)	Long-term (>1 year follow-up)	
	Delayed graft function	Graft failure at 1 year	Graft failure + death
**Recipient IL-18** Hall 2012 ^58^ Hall 2010 ^55^	OR 5.1 (1.14, 2.28)*	HR 1.26 (0.74, 2.14)	
**Recipient NGAL** Nauta 2011 ^59^ Parikh 2006 ^56^ Hall 2010 ^55^ Hall 2012 ^58^	OR 6.8 (1.42, 32.2)*	HR 1.61 (1.04, 2.50)	HR 1.5 (1.2, 1.8) HR 1.2 (1.0, 1.5) †
**Recipient NAG** Nauta 2011 ^59^			HR 1.4 (1.2, 1.7)
**Recipient KIM-1** Nauta 2011 ^59^			HR 1.3 (1.1, 1.7)
**Recipient H-FABP** Nauta 2011 ^59^			HR 1.5 (1.2, 1.8)

All values in ( ) are 95% confidence intervals, unless otherwise noted. *greater than cut-off *versus* less than cut-off, † per 100 ng/mg increase. AKI, acute kidney injury; H-FABP, heart fatty acid binding protein; HR, hazard ratio (continuous unless otherwise noted); IL-18, interleukin-18; KIM-1, kidney injury molecule-1; NAG, N-acetyl-beta-D-glucosaminidase; NGAL, neutrophil gelatinase-associated lipocalin; OR, odds ratio.

***Short-term outcomes.*** In the perioperative period, the presence of urinary IL-18 and NGAL immediately after transplant was associated with an increased risk of DGF ^55, 56^. While the relationship between recipient kidney injury biomarkers and outcomes is relatively clear, the relationship between donor kidney injury biomarkers and recipient outcomes is more complex. One large multi-center cohort found that elevated donor urinary NGAL had a modest association with DGF in the recipient, although other tubular injury biomarkers were not significantly associated after multivariable adjustment ^57^.

***Long-term outcomes.*** Single-center studies of transplant recipients found that elevated urinary tubular injury biomarkers, such as urinary IL-18, NGAL, NAG, and KIM-1, are associated with an increased risk of graft failure or death ( Table 4) ^58, 59^. However, urinary donor biomarkers, including urinary IL-18, NGAL, and L-FABP, modified the relationship between DGF and 6-month recipient GFR. For recipients with DGF, higher levels of tubular injury biomarkers were associated with higher 6-month GFR, while for recipients who did not experience DGF, higher urinary donor biomarkers were associated with lower 6-month GFR ^57^.

### Heterogeneous populations: hospitalized patients and the emergency department

While the most extensive evidence for the association between biomarkers of AKI and prognosis is in specific patient populations, biomarkers of AKI also have prognostic utility for short-term outcomes in heterogeneous populations. Multiple single-center cohorts of patients requiring nephrology consultation found that biomarkers of tubular injury were associated with an increased risk of in-hospital RRT or death ( Table 5) ^60– 63^. Alternatively, elevated plasma NGAL, a marker of glomerular filtration, in patients hospitalized with pneumonia and severe AKI meant they were twice as likely to fail to recover renal function by discharge in adjusted analysis ^64^. In another cohort of hospitalized patients requiring nephrology consultation owing to presumed acute tubular necrosis, elevated urinary epidermal growth factor levels allowed the authors to distinguish between patients who recovered renal function within 1 week of renal consult and those who did not. Additionally, elevated inflammatory cytokines identified patients who failed to recover renal function within 1 week of renal consult ^65^. Urinary NGAL and KIM-1 measured at the time of emergency room visit were both independently associated with in-hospital RRT initiation or death after accounting for clinical parameters ^66^.

**Table 5.** Biomarkers of AKI and outcomes in heterogeneous populations.

Biomarkers	IN-HOSPITAL OUTCOMES	
	RRT or death	Failure to recover from AKI
**Urine IL-18** Hall 2011 ^60^	OR 2.7 (1.4, 5.0)*	
**Urine NGAL** Hall 2011 ^60^ Singer 2011 ^63^ Nickolas 2011 ^66^	OR 2.6 (1.6, 4.3)* OR 4.2 (1.9, 9.0) § OR 2.43 (1.42, 4.16) §	
**Urine KIM-1** Liangos 2007 ^61^ Hall 2011 ^60^ Nickolas 2011 ^66^	AUC 0.61 (0.53, 0.69) OR 2.8 (1.5, 5.3)* OR 2.54 (1.50, 4.30) §	
**Urine NAG** Liangos 2007 ^61^	AUC 0.71 (0.63, 0.78)	
**Plasma NGAL** Srisawat 2011 ^64^		OR 2.02 (1.03, 3.31) †
**Urine EGF** Kwon 2010 ^65^		AUC 0.83 (0.70, 0.96)

All values in ( ) are 95% confidence intervals, unless otherwise noted. *4 ^th^ quartile *versus* 1 ^st^–3 ^rd^ quartile, § greater than cut-off *versus* less than cut-off, † per 300 ng/ml increase. AKI, acute kidney injury; AUC, area under the curve; EGF, epidermal growth factor; HR, hazard ratio; IL-18, interleukin-18; KIM-1, kidney injury molecule-1; NAG, N-acetyl-beta-D-glucosaminidase; NGAL, neutrophil gelatinase-associated lipocalin; OR, odds ratio; RRT, renal replacement therapy.

## Future directions

### Next-generation biomarkers

The bioinformatics revolution has improved discovery techniques for the identification of next-generation biomarkers. Biomarker research has primarily focused on biomarkers that are considered part of the proteome. Recent studies have begun examining other layers of cellular data, including microRNA and mitochondrial DNA ^67– 70^. While these biomarkers are in the early stages of investigation, they hold great promise, as they provide opportunities to probe other components of cellular pathways.

### Limitations for clinical application and future directions

Several barriers remain for the clinical application of biomarkers despite the extensive evidence that they are associated with prognosis. Biomarkers of AKI often provide modest improvement in discriminative ability compared to traditional clinical models and have different trajectories and time scales depending upon the patient population and biomarker being studied. This will pose a challenge for busy clinicians attempting to incorporate a cornucopia of biomarkers into clinical practice. Moreover, data are lacking to support whether biomarkers are associated with organ-specific long-term outcomes, such as CKD or cardiovascular disease. Technical challenges also exist, as developing, standardizing, and validating biomarker assays is a laborious process ^71^. Most importantly, researchers have yet to identify therapeutic changes to institute once patients are identified as being at high risk for adverse outcomes based on biomarkers of AKI.

Given the heterogeneity and dynamic nature of AKI, one biomarker will not accurately predict all adverse outcomes across several settings, nor will it accurately identify the various stages in the natural history of AKI. One important task is to definitively identify the natural stages of AKI as demarcated by biomarkers: injury, maintenance, and repair. This could help create a more balanced picture of prognostic outcomes for patients. For example, patients may have different outcomes depending on the magnitude of “injury” biomarkers balanced by the level of “repair” biomarkers.

While challenges remain, biomarkers can offer several potential benefits to the diagnosis, management, and treatment of AKI in the foreseeable future. Perhaps the most immediate application is to identify patients who are experiencing renal injury from nephrotoxic agents, prolonged surgeries, cardiorenal syndrome, or hepatorenal syndrome. Patients with elevated injury or fibrosis markers could be considered for alternative therapy instead of waiting for these patients to develop AKI or CKD as defined by serum creatinine. However, for biomarkers to become the “standard of care” for the management of these patients, prospective trials should be conducted to identify cut-points for biomarker levels which will identify patients at a sufficiently high risk of renal complications to warrant providing a therapeutic intervention or adjusting surgical management.

Biomarkers of AKI can have two potential applications in clinical trials of putative drug treatments for AKI. First, they will allow us to identify patients who have ongoing injury so that therapies may be provided while the injury process is occurring. Second, the greatest potential for biomarkers is if they can identify patients at high risk of adverse outcomes. In such a scenario, treatment can then be personalized for high-risk subgroups of patients. Trials in AKI have been hindered by low event rates and are likely enrolling patients with multiple phenotypes of AKI. Given the wealth of information showing that biomarkers are related to important patient outcomes, such as RRT and death, future clinical trials for AKI should enroll patients with elevated biomarkers of AKI in order to increase the likelihood that patients would actually benefit from the putative treatment. The TOPCAT trial, which examined the benefit of spironolactone in heart failure patients with preserved ejection fraction, found that treatment benefit was isolated to patients with elevated natriuretic peptide levels ^72^. Future AKI trials should employ similar enrollment strategies, as treatment benefit may be isolated to particular AKI phenotypes. The Assessment, Serial Evaluation and Subsequent Sequelae of Acute Kidney Injury study is ongoing to determine if patients with AKI experience increased risk of long-term complications, such as cardiovascular events and CKD ^27^. This study should help ascertain if it is worthwhile enrolling patients with AKI as defined by biomarkers in lieu of serum creatinine in future clinical trials.
